# 741 Burn Injury Elevates the Risk of Sepsis in Pregnant Women

**DOI:** 10.1093/jbcr/irac012.294

**Published:** 2022-03-23

**Authors:** Shelby P Bagby, Juquan Song, Kendall Wermine, George Golovko, Amina El Ayadi, Steven E Wolf

**Affiliations:** University of Texas Medical Branch, Galveston, Texas; University of Texas Medical Branch, Galveston, Texas; University of Texas Medical Branch, Galveston, Texas; University of Texas Medical Branch at Galveston, Galveston, Texas; University of Texas Medical Branch at Galveston, Galveston, Texas; University of Texas Medical Branch at Galveston, Galveston, Texas

## Abstract

**Introduction:**

Pregnancy naturally strains a woman’s body, and is exacerbated by additional stressors, such as severe burn. This study seeks to establish a national incidence rate of burns during pregnancy, as well as categorize the patients epidemiologically and by percent total body surface area (%TBSA) burned. We posit that pregnancies complicated by burn injuries have worse outcomes in mortality and comorbidities in comparison to pregnancies not complicated by burns.

**Methods:**

Using an electronic medical record database, TriNetX, a retrospective cohort study was performed to identify burned pregnant patients within the last 20 years. The burn cohort included all pregnant women aged 12-55 who experienced a burn injury on the same day as pregnancy or anytime within nine months after the first record of pregnancy. The non-burned cohort included women who did not experience a burn within nine months of the recorded pregnancy. Outcomes compared were sepsis, pregnancy with abortive outcome, ectopic pregnancy, spontaneous abortion, complications of labor and delivery, preterm labor, postpartum hemorrhage, maternal mortality, and acute respiratory distress syndrome (ARDS). After matching for age at pregnancy, each outcome was compared at one, three, and five years after pregnancy. Risk ratios (RR) with a 95% confidence interval (CI) were used to compare cohorts, and a p-value < 0.05 was deemed significant.

**Results:**

The TriNetX database contained 21,438,975 females between the ages of 12-55. Among these, pregnant women with burn injuries were found to have an incidence of 4.32% in the United States in the last 20 years (pregnant females with burn n = 4,721; females with burn n = 109,294). Of burns categorized by %TBSA burned, 84% were between 1-10%. Within one year of pregnancy, burned patients have a three-fold increase in risk of development of sepsis compared to non-burned women (RR = 3, 95% CI = 1.518, 5.929), but are less likely to experience pregnancy with abortive outcome (RR = 0.612, 95% CI = 0.509, 0.735), complications during labor and delivery (RR = 0.863, 95% CI = 0.803, 0.928) or spontaneous abortion (RR = 0.707, 95% CI = 0.556, 0.899).

**Conclusions:**

Pregnancy complicated by burn injury has a lower national incidence rate than the generally accepted 7% of reproductively aged females. Burned patients were more likely to experience sepsis than their non-burned counterparts one year after pregnancy, however, risk of maternal mortality was the same between the burned and non-burned patients within one year after pregnancy with a curious decrease in miscarriage and labor and delivery complications.

